# A patient with *TPCN2*-related hypopigmentation and ocular phenotype

**DOI:** 10.1038/s41431-024-01779-5

**Published:** 2025-01-14

**Authors:** Cécile Courdier, Vincent Michaud, Modibo Diallo, Claudio Plaisant, Eulalie Lasseaux, Isabelle Helot, Elodie Philippe, Els Vrielynck, Marjolaine Willems, Benoit Arveiler

**Affiliations:** 1 Service de Génétique Médicale, Centre Hospitalier Universitaire de Bordeaux, Bordeaux, France; 2 Laboratoire Maladies Rares, Génétique et Métabolisme, Bordeaux University INSERM U1211, Bordeaux, France; 3 Centre d’ophtalmologie VISIS, Espace Méditerranée, 66000 Perpignan, France; 4 Service de Génétique Clinique, Centre Hospitalier Universitaire de Montpellier, Montpellier, France; 5 Present Address: Institut Bergonié, Bordeaux, France

**Keywords:** Disease genetics, Next-generation sequencing

## Abstract

Pigmentation is orchestrated by hundreds of genes involved in cellular functions going from early developmental fate of pigment cells to melanin synthesis. The Two Pore Channel 2 (TPC2) a Ca2+ and Na+ channel acidifies melanosomal pH and thus inhibits pigmentation. A young patient was recently reported with generalized hypopigmentation but uneventful ocular examination, caused by the de novo heterozygous *TPCN2* variant c.628C>T;p.Arg210Cys that constitutively activates TPC2. Here we report a young patient with the same de novo variant presenting with generalized hypopigmentation, and ophthalmologic features including low grade retinal hypopigmentation and foveal hypoplasia, photophobia, mild hypermetropia, and astigmatism, which are features of albinism. Skin fragility and episodes of fever with diarrhea and fatigue were also observed. This extends the phenotype of patients with *TPCN2* variants, warranting further investigations in patients with alterations of this gene, and raises the question whether *TPCN2* might be considered as an albinism gene.

## Introduction

Pigmentation is a complex process that requires the involvement of hundreds of genes and proteins, as compiled by Baxter et al. [1]. These genes encode proteins with a wide range of cellular functions going from pigment cell differentiation and migration at early developmental stages to the correct functioning of pigment cells. These are melanocytes in the skin, hair and choroid, and retinal pigment epithelial cells. Melanin synthesis takes place in lysosome-related organelles called melanosomes, which undergo maturation from immature stage 1 to mature melanin-loaded stage 4. It requires protein complexes involved in melanosomal structure and maturation and in the transport of cargo, melanogenic enzymes (tyrosinase, tyrosinase related protein 1, and dopachrome tautomerase), and ion channels necessary for homeostasis of Na+, Ca2+, Cl− and pH regulation [2]. Indeed maintaining a neutral pH is critical since acidification is detrimental for the catalytic activity of tyrosinase. Dysfunction of proteins at any stage of this complex process can lead to pigmentation disorders such as piebaldism, Waardenburg syndrome and albinism [1].

Among the ion channel and transporters genes involved in pigmentation disorders three are altered in subtypes of oculocutaneous albinism (OCA) (*OCA2*, *SLC45A2*, and *SLC24A5*, in OCA 2, 4 and 6 respectively) [3, 4]. A dominant variant of the Cl- channel *CLCN7* gene has been described in two unrelated patients with hypopigmented skin and hair, in the context of a more complex phenotype [5].

Two Pore Channel 2 (TPC2) is a Ca2+ and Na+ channel that has been localized to melanosomes and endolysosomes and is activated by PI(3,5)P2. TPC2 acidifies melanosomes and inhibits melanin synthesis [6]. Instead, loss-of-function of TPC2 alkalizes melanosomes and promotes pigmentation [7]. Of note, TPC2 variants p.Met484Leu and p.Gly734Glu are associated with lighter hair color in Northern Europeans [8] due to their gain-of-function properties [9]. Böck et al. [10] identified additional common variants and showed that p.Leu564Pro is the predominant TPC2 variant and is crucial for the blond hair-associated p.M484L gain-of-function effect.

Recently, Wang et al. [11] described a young Chinese girl carrying a de novo heterozygous variant, NM_139075.4:c.628C>T;p.Arg210Cys in the *TPCN2* gene that encodes TPC2. The patient exhibited hypopigmentation of both hair and skin but had uneventful ocular examinations, with normal visual acuity, normal macula and fovea, normal retinal pigmentation and absence of nystagmus and photophobia. Arginine 210 is located in the S4-S5 linker containing a poly-basic amino acid region which interacts with the TPC2 agonist PI(3,5)P2. The Arg210Cys variant results in constitutive activation of TPC2, characterized by increased affinity for PI(3,5)P2, increased Ca2+ release from and H+ entrance in the lysosomal lumen. Since TPC2 also localizes to the melanosome, the authors assumed that a similar effect would be found in these lysosome-related organelles. In addition, mice harboring the homologous mutation Arg194Cys also exhibit hypopigmentation in the fur and skin, as well as less pigment and melanosomes in the retina. Altogether, these data indicate that the Arg210Cys variant likely acts as a gain-of-function variant explaining hypopigmentation of skin and hair, in agreement with the previous findings that TPC2 mild gain-of-function variants, p.Met484Leu and p.Gly734Glu, are associated with lighter skin and hair colors in Northern Europeans.

## Materials and methods

Albinism panel sequencing was performed as described in Diallo et al. [12].

Exome sequencing: Library preparation and exome capture were performed using SureSelect Human All Exon V8 kit (Agilent), followed by paired-end 75 bases massively parallel sequencing on Illumina NextSeq 550Dx (Illumina).

Sanger sequencing was performed using the Big Dye Terminator v3.1 kit on an ABI3500xL Dx (Thermo Fisher Scientific).

The NM_139075.4:c.628C>T;p.Arg210Cys *TPCN2* variant was submitted to ClinVar (submission number SCV005397923).

## Results

### Clinical description

**Fig. 1:**
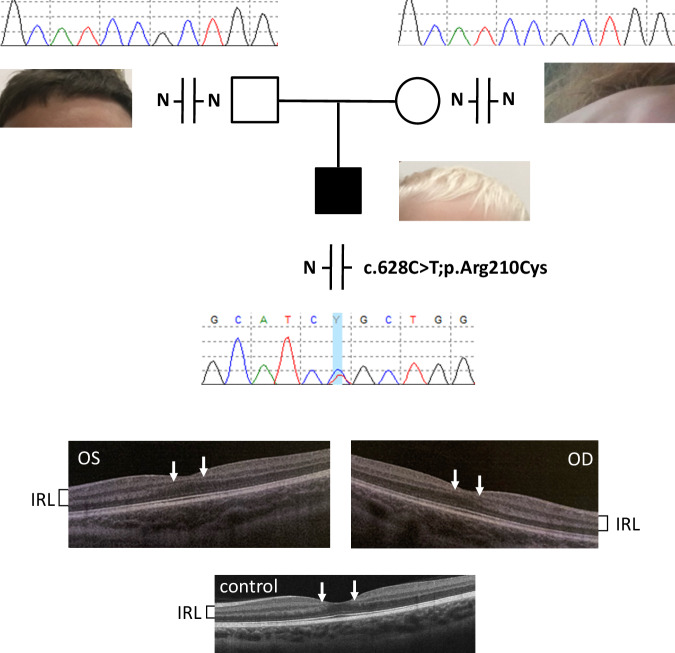
Pedigree of the family. Genotypes are indicated for each family member, next to vertical bars representing each allele. N: normal allele. Electrophoregrams displaying the Sanger sequence encompassing *TPCN2* variant NM_139075.4:c.628C>T;p.Arg210Cys are provided for each family member. The blue vertical bar indicates the presence of the variant in the heterozygous state in the patient. Photographs showing skin and hair color of the patient and his parents are provided. Optical coherence tomography (OCT) images of the patient is shown underneath (OD oculus dextra, OS oculus senestra). Arrows on both sides of the foveal pit point to the inner retinal layers that show intrusion in the foveal region in both eyes in the patient, whereas they are extruded from the foveal region in the healthy control. Inner retinal layers (IRL) are also indicated by brackets on the side of the images. OCT image of a healthy control is displayed at the bottom.

We report a French 2 years old male patient presenting with generalized hypopigmentation of the hair and skin, who was referred to our laboratory for molecular diagnosis of albinism. At birth he had white skin (phototype 0) and hair, and gray eyes. At the age of two his skin and hair remain unpigmented, and eyes are greyish with light blue-green shades (Fig. 1). Freckles appeared at about 20 months. Five small naevi (1–3 mm in diameter) are observed. His skin is extremely fragile, as it marks easily at contact with textiles, resulting in the appearance of red patches and spots. On injury, there is no excessive bleeding but healing takes several months.

At the ocular level the patient presents with photophobia but no nystagmus. Cycloplegic refraction shows mild hypermetropia with astigmatism in both eyes. Spherical equivalent is of +1.00 diopter and +1.25 in right and left eye, respectively. Cylinder power is of 0.50 diopter in both eyes. No iris transillumination is noted. Fundus examination shows mild retinal hypopigmentation (grade 1). Grade 1 foveal hypoplasia is observed by optical coherence tomography on both eyes showing intrusion of inner retinal layers in the foveal region in both eyes. On the contrary, extrusion of inner retinal layers of the retina is observed in the foveal region of a healthy control (see Fig. 1). Visual acuity is 10/10 on both eyes, and 10/10 binocular.

Psychomotor development is normal.

The patient’s mother reports episodes of fever with diarrhea and fatigue that last for one day, followed by body-wide skin eruptions that start ceding after 3 days.

The patient’s mother has white skin and brown eyes. The father has light brown skin, hair and eyes.

### Molecular analysis

Sequencing of the 21 known albinism genes using our next generation sequencing panel [3, 12] identified a heterozygous variant in the *HPS1* gene, NM_000195.5:c.1718C>G, p.Pro573Arg. This variant was classified as a variant of unknown significance according to Richards et al. [13]. No other variant was identified in *HPS1* despite complete sequencing of all exons and introns of this gene. Platelet functional investigations did not show any defect associated with Hermansky-Pudlak syndrome. These data therefore exclude HPS1 in the patient. No other pathogenic variant was observed in the other albinism genes.

Whole exome sequencing of the patient and his parents was then performed. The same *TPCN2* variant as that identified in [11], NM_139075.4:c.628C>T;p.Arg210Cys, was found in the heterozygous state, de novo, in the patient (Fig. 1).

## Discussion

Interestingly, whereas the patient described by Wang et al. [11] did not present with an ocular phenotype, our patient presents with grade 1 retinal hypopigmentation, photophobia and grade 1 foveal hypoplasia, hypermetropia and astigmatism. He also has noticeable phenotypic features such as skin fragility and tendency to develop red patches and spots, slow healing, and episodes of fever with diarrhea and fatigue followed by body-wide skin eruption, which were not reported by Wang et al. [11] in their patient, nor in the orthologous mouse model they made. The symptoms may however not be related to the *TPCN2* variant, but be signs of another disease. However, exome sequencing did not identify any specific variant that could account for these symptoms. It will therefore be important to study more patients with this and other pathogenic variants of *TPCN2* in order to further delineate the associated phenotype. Of note, mice heterozygous for the orthologous variant Arg194Cys showed some degree of retinal hypopigmentation [11].

Whereas gain of function *TPCN2* variants are responsible for decreased melanogenesis, loss of function of this gene was shown to alkalize melanosomes and promote pigmentation in human and mouse cultured melanocytes [6, 7]. While no *TPCN2* loss of function variants were described so far in humans, it will be interesting to see their effect at both the cutaneous and ocular levels once they will be identified.

Whether *TPCN2* might be considered an albinism gene is an open question. Albinism corresponds to a wide spectrum of phenotypes, associating variable degrees of hypopigmentation ranging from very severe to mild or even normal, and ocular features including nystagmus, retinal hypopigmentation, iris transillumination, foveal hypoplasia, photophobia and reduced visual acuity. The phenotypical severity depends upon the gene involved and the type of variants present in the patient [3, 4, 14, 15]. Intriguingly, while OCA 4 is commonly associated with classical ocular features of albinism, some OCA 4 patients have generalized hypopigmentation but no ocular features [16, 17].

All forms of albinism described so far are recessive (autosomal for 20, X-linked for 1) [3, 4]. It has however been reported that 30% of heterozygous parents, although not clinically diagnosed with the disease, have mild clinical signs [18, 19], thus raising the possibility for the existence of dominant forms of albinism. One example of dominant melanogenesis defect is that caused by a heterozygous gain of function variant of the chloride channel gene *CLCN7* [5].

*TPCN2* c.628C>T;p.Arg210Cys constitutes the second example of a dominant gain of function variant responsible for generalized hypopigmentation, associated with some albinism-related ocular features in one case (reported here), or not associated with ocular features in one case [11]. The description of additional patients harboring this or other variants of *TPCN2* will be important to better define the clinical phenotype of *TPCN2*-related disease. Considering the wide phenotypic spectrum of albinism, it is possible that *TPCN2* will become a new albinism gene, opening a new chapter of “patients with generalized hypopigmentation but no or little ocular features”.

### Variant validator nomenclature check

Transcript NM_139075.4:c.628C>T

Protein three letter code NP_620714.2:p.(Arg210Cys).

## Data Availability

All data are available from the authors upon request.
